# Complementing the Cancer-Immunity Cycle

**DOI:** 10.3389/fimmu.2019.00774

**Published:** 2019-04-12

**Authors:** Ruben Pio, Daniel Ajona, Sergio Ortiz-Espinosa, Alberto Mantovani, John D. Lambris

**Affiliations:** ^1^Program in Solid Tumors (CIMA) and Department of Biochemistry and Genetics (School of Medicine), University of Navarra, Pamplona, Spain; ^2^Navarra Institute for Health Research (IDISNA), Pamplona, Spain; ^3^Centro de Investigación Biomédica en Red de Cáncer (CIBERONC), Madrid, Spain; ^4^Humanitas Clinical and Research Center, Humanitas University, Milan, Italy; ^5^William Harvey Research Institute, Queen Mary University of London, London, United Kingdom; ^6^Department of Pathology and Laboratory Medicine, University of Pennsylvania, Philadelphia, PA, United States

**Keywords:** cancer immunity, immunotherapy, complement system, C3a, C5a, C1q, PD-1, PD-L1

## Abstract

Reactivation of cytotoxic CD8^+^ T-cell responses has set a new direction for cancer immunotherapy. Neutralizing antibodies targeting immune checkpoint programmed cell death protein 1 (PD-1) or its ligand (PD-L1) have been particularly successful for tumor types with limited therapeutic options such as melanoma and lung cancer. However, reactivation of T cells is only one step toward tumor elimination, and a substantial fraction of patients fails to respond to these therapies. In this context, combination therapies targeting more than one of the steps of the cancer-immune cycle may provide significant benefits. To find the best combinations, it is of upmost importance to understand the interplay between cancer cells and all the components of the immune response. This review focuses on the elements of the complement system that come into play in the cancer-immunity cycle. The complement system, an essential part of innate immunity, has emerged as a major regulator of cancer immunity. Complement effectors such as C1q, anaphylatoxins C3a and C5a, and their receptors C3aR and C5aR1, have been associated with tolerogenic cell death and inhibition of antitumor T-cell responses through the recruitment and/or activation of immunosuppressive cell subpopulations such as myeloid-derived suppressor cells (MDSCs), regulatory T cells (Tregs), or M2 tumor-associated macrophages (TAMs). Evidence is provided to support the idea that complement blocks many of the effector routes associated with the cancer-immunity cycle, providing the rationale for new therapeutic combinations aimed to enhance the antitumor efficacy of anti-PD-1/PD-L1 checkpoint inhibitors.

## Introduction

Profound advances in our understanding of the interactions between tumors and the immune system have allowed the development of therapeutic approaches that boost the body's natural defenses against cancer. These therapies are aimed to mount effective antitumor immune responses and include immunomodulators, vaccines, and adoptive transfer of immune cells (1). Some of the most clinically effective immunotherapies to date target the programmed cell death protein 1 (PD-1) immune checkpoint. PD-1 is expressed by T cells during priming or expansion and binds to one of its two ligands PD-L1 or PD-L2 (2). Tumor cells upregulate PD-L1 in response to cytokines such as interferon (IFN)-γ (3). Interaction of PD-L1 with PD-1 on T cells causes T-cell apoptosis, anergy, and exhaustion, protecting tumor cells from CD8^+^ T cell-mediated cytolysis (3). PD-L1 can also deliver intrinsic intracellular signals that enhance cancer cell survival, regulate stress responses, and confer resistance toward apoptotic stimuli (4, 5). PD-1 and other checkpoints are also expressed by NK cells which may contribute to the antitumor activity of some therapeutic strategies under development (6, 7).

Neutralizing monoclonal antibodies against PD-1 or PD-L1 have transformed the therapeutic landscape of a wide range of cancers (Table 1), being particularly successful for tumors with limited therapeutic options such as melanoma or lung cancer (3). Notably, these antibodies generate durable responses without causing serious side effects. However, a significant fraction of patients manifests innate or acquired resistance to these therapies. Immune escape mechanisms stem from different cell interactions within the tumor microenvironment, and emphasize the need of developing rational combination strategies to obtain more potent anticancer responses (8).

**Table 1:** FDA-approved immune-checkpoint inhibitors (monoclonal antibodies) for cancer treatment.

**Drug**	**Brand name**	**Target**	**Antibody subclass**	**Cancer type**
Nivolumab	Opdivo	PD-1	Human IgG4	Melanoma Non-small cell lung cancer Small cell lung cancer Renal cell cancer Hodgkin lymphoma Head and neck cancer Urothelial cancer Colorectal cancer^*^ Hepatocellular cancer
Pembrolizumab	Keytruda	PD-1	Humanized IgG4	Melanoma Non-small cell lung cancer Head and neck cancer Hodgkin lymphoma Primary mediastinal B-cell lymphoma Urothelial cancer Solid tumors^*^ Gastric cancer Cervical cancer
Atezolizumab	Tecentriq	PD-L1	Humanized IgG1	Urothelial cancer Non-small cell lung cancer
Durvalumab	Imfinzi	PD-L1	Human IgG1	Urothelial cancer Non-small cell lung cancer
Avelumab	Bavencio	PD-L1	Human IgG1	Merkel cell cancer Urothelial cancer

* *For patients with mismatch repair deficiency (dMMR) or microsatellite instability high (MSI-H)*.

In 2013, Daniel Chen and Ira Mellman described a series of self-sustaining stepwise events, referred as the cancer-immunity cycle, by which the anti-cancer immune responses lead to an effective elimination of cancer cells (9). The existence of negative feedback mechanisms developed by tumors hinders this cycle of cancer immunity and may pose a barrier to the development of effective clinical responses. Anticancer immunotherapies should be aimed to reactivate all the steps of the cycle, which include immunogenic cell death, maturation of antigen-presenting cells, T-cell priming and activation, promotion of immune infiltration, blockade of immunosuppression, and enhancement of effector T-cell activity. In this context, combination therapies would provide synergistic effects for the maintenance of the cancer-immunity cycle.

In the last years, the complement system, an essential part of innate immunity, has surged as a master regulator of cancer immunity (10). We, and others, have actively contributed to this field, leading to the proposal that modulation of complement activation can improve the antitumor efficacy of inhibitors targeting the PD-1/PD-L1 pathway. In 2008, making a paradigm shift in tumor immunology, we demonstrated that complement activation, followed by C5a signaling, has a tumor-promoting role in cancer (11). In 2012, using a lung cancer model, we first demonstrated an association between the inhibition of C5a receptor 1 (C5aR1) and the expression of PD-L1 within the tumor microenvironment (12). These results suggested the possibility of blocking complement factors to increase the efficacy of other immune therapeutic strategies (12). Following this line, we demonstrated that inhibition of PD-1/PD-L1 synergizes with the inhibition of C5a/C5aR1 in various preclinical models of lung cancer (13). This rationale provided the basis for a clinical trial in which the anti-C5aR1 antibody IPH5401 is being evaluated in combination with the anti-PD-L1 antibody durvalumab in patients with solid tumors (STELLAR-001).

In this review we describe the participation of complement elements in the steps of the cancer-immunity cycle. We propose that a combinational therapy using anti-PD-1/PD-L1 antibodies together with modulators of the complement system may open new therapeutic opportunities for tumors resistant to PD-1/PD-L1 blockade.

## The Cancer-Immunity Cycle

The cancer-immunity cycle is defined as a series of functional stepwise events needed to obtain an efficient control of cancer growth by the immune system (9). The process is initiated by the release of neo-antigens generated as a result of genomic instability. Cancer-associated antigens are captured by dendritic cells which, upon migration to lymph nodes, prime and activate tumor-specific cytolytic CD8^+^ T cells. These effector cells migrate and infiltrate the tumor stroma, where potentially are able to recognize and eliminate cancer cells. T cell-mediated cytotoxic responses release new tumor antigens, fueling the cancer-immunity cycle. Interestingly, this model provides the rationale for targeting different steps of the cycle in order to maintain its functionality. An effective cancer immunotherapy should be designed based on the specific resistance mechanisms underlining the rate-limiting steps in each particular patient (14, 15). In the case of anti-PD-1/PD-L1 therapies, a variety of biological factors contribute to treatment resistance, including lack of cancer antigens recognizable by T cells, impaired cancer-antigen presentation, impaired activation of cancer-specific T cells, poor infiltration of T cells into tumors, and accumulation of immunosuppressive factors and cells in the tumor microenvironment (15). The evading strategies present in a given tumor would determine whether this tumor shows an inflamed or a noninflamed phenotype (16). Clinical evidence suggests that anti-PD-1/PD-L1 inhibitors are most effective in inflamed tumors characterized by high tumor PD-L1 expression, CD8^+^ T-cell infiltration or mutational burden (17–20). Jerby-Arnon et al. recently analyzed the relationship between malignant cell states and CD8^+^ T-cell infiltration and identified a T-cell exclusion program that predicts responses to PD-1/PD-L1 blockade (21). This program was enriched for genes involved in predictable processes, such as antigen processing and presentation, IFN-γ signaling, and immune modulation; but also identified genes associated with activation and modulation of the complement system (21). Elements of the complement cascade are also present in a signature of serum proteins that predicts survival in patients receiving PD-1 blocking antibodies, suggesting that complement activation may inhibit the efficacy of adaptive antitumor immunity (22).

## The Complement System

The complement system, a central element of innate immunity, represents a first line of defense against unwanted non-self and host elements, and orchestrates many immunological and inflammatory processes that substantially contribute to body homeostasis (23, 24). Complement activities are mediated by more than 50 circulating, cell surface-bound and intracellular proteins. There are three main mechanisms of complement activation, known as classical, lectin, and alternative pathways. The classical pathway is commonly initiated by the binding of C1q to complement-fixing antibodies (mostly IgM and IgG types); although C1q can also recognize non-immunoglobulin ligands such as C-reactive protein (CRP), pentraxin 3 (PTX3), or apoptotic cells (25). The lectin pathway is activated by homologous proteins to C1q (mannose-binding lectin, collectins, and ficolins) that recognize repetitive carbohydrate patterns (26). Lastly, the alternative pathway is initiated by spontaneous cleavage of C3 on activating surfaces (27). Although the three complement pathways differ in their mechanisms of target recognition, in all cases, initiation of the complement cascade leads to the formation of C3 convertases and the activation of the central component C3. After this activation, C5 convertases are formed, C5 is cleaved, and the assembly of the pore-like membrane attack complex (MAC) is initiated. The enzymatic cleavage of complement elements leads to the release of proteolytic fragments such as C3a and C5a, and the deposition of other fragments such as C3b and iC3b. These molecules modulate a diverse set of processes (23), including the initiation and regulation of effector T-cell responses (28). Prevention of inappropriate activation by complement regulators takes place at three main levels: inhibition of protease activities in the activation cascade, decay and destruction of convertases, and control of MAC formation (29). Finally, recent experimental and clinical evidences suggest that intracellular complement components have important roles in cell physiology (30).

Complement has been traditionally regarded as playing a role in the elimination of tumor cells. Accordingly, an effective control of tumor growth may be achieved by complement-fixing antibodies (31). However, growing evidence, starting for the initial observation in a model of cervical cancer (11), strongly supports a tumor-promoting role of complement in several tumor types. This topic has been extensively reviewed elsewhere (10, 32–35). Briefly, complement establishes an immunosuppressive microenvironment, promotes angiogenesis, sustains cellular proliferation, and participates in tumor cell invasion and migration. In light of the various contributions of complement to cancer progression, it is not surprising that expression of complement effectors and receptors is associated with disease progression and poor prognosis (36–42). Among all the complement elements with potential pro-cancer activities, C1q, C3-derived fragments, and C5a are recognized as major modulators of tumor progression (43–45).

## Complement in the Regulation of the Cancer-Immunity Cycle

In this section we will discuss the potential implication of effectors and regulators of the complement system in the steps of the cancer-immunity cycle. In light of the breadth and complexity of the immune response, we will focus our review on the specific aspects of the regulation of CD8^+^ cytotoxic T cells, which are in large part the mediators of anti-PD-1/PD-L1 therapies. We will also examine evidence supporting the participation of complement in the regulation of the type 1 T helper (Th1) response, as it has a profound influence on the quality and extension of cytotoxic T-cell responses (46). Recent reviews have extensively addressed other complement-mediated immune functions not covered in the present review (10, 28, 47–50).

### Modulation of the Initiation of T-Cell Immunity by the Complement System

The cancer-immunity cycle is initiated by tumor-specific neo-antigens generated by somatic mutations (51). Dying cancer cells release these antigens to the tumor microenvironment, where are captured and processed by dendritic cells, the principal cell type responsible for instructing naïve T cells to undergo antigen-specific effector functions. Depending on the stimuli provided by dying cancer cells, their interaction with dendritic cells can have immunogenic or tolerogenic consequences (52). The generation of an immunogenic or a tolerogenic cell death is mainly regulated by damage-associated molecular patterns (DAMPs). DAMPs are endogenous co-stimulatory signals secreted or presented on the cell surface of dying cells that interact with pattern-recognition receptors (PRRs) alerting the host of danger. Complement is required for efficient sensing of DAMPs (53–55). The specific interactions of danger sensors with complement elements allow to differentiate between physiological and pathological danger, shaping the maturation of dendritic cells (23). This activity depends mainly on the classical complement pathway. Direct binding of complement C1q to apoptotic cells promotes a phagocytic-mediated uptake of dying cells, which sustains an anti-inflammatory innate immune response through the expression of cytokines such as transforming growth factor (TGF)-β (56). In fact, genetic deficiencies in C1q, as well as other elements of the classical complement pathway, can compromise the induction of self-tolerance and result in systemic autoimmune diseases (57, 58). Another complement element involved in the recognition of danger signals is factor H, a soluble complement inhibitor produced and secreted by cancer cells (59, 60). Upon opsonization of apoptotic cells, factor H induces an anti-inflammatory cytokine profile (61, 62) and a tolerogenic stage (63). CD46, a membrane-bound complement regulatory protein able to interact with C3 activation fragments and found at high levels in some cancer types (64, 65), has also been proposed as a negative regulator of immune recognition (66). Complement proteins are easily detectable in various types of cancer, consistent with complement activation by these tumors (32). Therefore, upregulation of complement components in the surface of dying cancer cells may be associated with a tolerogenic cell death, in contrast to the immunogenic cell death required for an effective anticancer immune response (67, 68).

### Modulation of Priming and Activation of T Cells by the Complement System

Progress of the cancer-immunity cycle requires the presence of activation signals that allow dendritic cells to mature, migrate to the lymph nodes, and present the neo-antigens to naïve T cells. Efficient priming also relies on the contextual information provided by the microenvironment. Mature dendritic cells in the presence of suitable signals are able to induce T-cell effector functions; whereas in the absence of appropriate conditions, antigen presentation leads to T-cell anergy or generation of regulatory T cells (Tregs) that suppress effector responses.

Locally-produced complement elements determine the state of dendritic cell activation (69), and are critical in the regulation of T-cell responses (28). Production of C1q and C3 by dendritic cells induces their maturation and their capacity to stimulate Th1-cell responses (70, 71). C3 may also facilitate intracellular antigen processing and presentation (72). In agreement with these observations, optimal priming and expansion of CD4^+^ and CD8^+^ T cells in infection models is dampened by C3 deficiency (73, 74), and the complement fragment C3d amplifies antitumor T-cell responses (75). In the case of C3a and C5a, through activation of their respective receptors C3aR and C5aR1, these anaphylatoxins enhance the capacity of human monocyte-derived dendritic cells to stimulate T cells (76). In accordance, C3aR pathway inhibition in dendritic cells results in defective T-cell priming, associated with a reduced surface expression of major histocompatibility complex (MHC) and costimulatory molecules (77). Finally, downregulation of the expression of the complement regulator CD55 in antigen-presenting cells during T-cell activation increases the local production of C3a and C5a, providing costimulatory signals to induce T-cell proliferation and differentiation (78, 79).

Complement elements can also exert a direct influence on T cells. Activation of human CD4^+^ T cells by CD46 stimulates the effector potential of Th1 cells (80–82). As on dendritic cells, paracrine and autocrine interactions of C3a and C5a with their respective receptors C3aR and C5aR1 mediate Th1 cytokine production and T-cell induction (78, 83). It has also been suggested that Tregs express C3aR and C5aR1, and that signaling through these receptors inhibits Treg function (84, 85).

A central role in T-cell homeostasis has been recently assigned to intracellular elements of the complement system (30). Activation of lysosomal C3aR by intracellularly generated C3a contributes to the survival of resting CD4^+^ T cells. Upon activation, the intracellular stores of the C3 system translocate to the cell surface triggering the upregulation of IFN-γ and Th1-cell responses in conjunction with the extracellular engagement of C3b to CD46 (86). Intracellular C5a can also be generated from endogenous C5. Upon T-cell activation, C5a binds to intracellular C5aR1, inducing the activation of the NLRP3 inflammasome and, consequently, the initiation of a Th1 response (87).

All these activities underline the importance of complement effectors and regulators in the initiation of T-cell responses. In contrast, the complement system has also been associated with the prevention of T-cell priming and the induction of tolerance; probably as a regulatory mechanism to facilitate the timely resolution of the immune response. Thus, C1q can suppress macrophage-mediated inflammation and dendritic cell-mediated Th1-cell proliferation (88, 89). Binding of the C3 fragment iC3b to complement receptor type 3 (CR3) on antigen-presenting cells results in the production of TGF-β2 and interleukin (IL)-10, and the induction of antigen-specific tolerance (90). C3a and C3b also participate in the contraction phase of human Th1 responses (80, 82). CD46 promotes the switching of CD4^+^ T cells toward IL-10 producing cells with a regulatory phenotype (80, 91), and negatively regulates Th1 activity through the binding of endogenous C5a to surface-expressed C5aR2 (87). C5aR2 was first proposed as a negative regulator of C5aR1, but some specific functions have been ascribed to this C5a receptor (92). CD55 may also have a role in the suppression of adaptive immune responses. Mice lacking CD55 experience enhanced T-cell responses to active immunization, characterized by an increased production of INF-γ and IL-2, as well as downregulation of IL-10 (93).

All these studies point to the dual role played by complement in the activation of effector T-cell responses. On the one hand, complement elements are central in the primary phase of effector expansion. On the other hand, complement can mediate a suboptimal T-cell activation associated with the contraction phase or the establishment of tolerance. With this duality in mind, it is interesting to analyze the relative role played by complement in the context of well-established tumors. It has been suggested that C5a affects T-cell responses in a concentration-dependent manner (94). Tumor-bearing mice with low C5a-producing tumor cells exhibit a reduced tumor burden with increased IFN-γ-producing CD4^+^ and CD8^+^ T cells in the spleen and tumor-draining lymph nodes. In contrast, tumor-bearing mice with high C5a-producing cancer cells have an accelerated tumor progression with less CD4^+^ and CD8^+^ T cells in the tumor, tumor-draining lymph nodes, and the spleen (94). This effect was associated with the presence of more myeloid-derived suppressor cells (MDSCs) in the spleen. Interestingly, other studies have found elevated levels of C5a in cancer patients (12), which have been implicated in the recruitment of MDSCs to tumors (11). MDSCs are immunosuppressive immature myeloid cells able to disrupt major mechanisms of antitumor immune responses (95–97). In models of breast cancer, C5aR1 signaling in MDSCs induces the production of immunosuppressive cytokines, such as TGF-β, and reduces Th1 immune responses (98, 99). Treatment of mouse squamous cell carcinomas with paclitaxel and PMX-53, a C5aR1 inhibitor, results in peripheral priming and expansion of antigen-specific clones (100). Therefore, we can conclude that complement-mediated effects may have evolved at established tumors to interfere with the generation of antitumor T-cell responses.

### Modulation of T-Cell Trafficking by the Complement System

A range of tumors escape from antitumor immune responses even after activation and expansion of T cells. In some cases, this can be attributed to the ability of the tumor endothelium to prevent T-cell trafficking. A complex network of endothelial adhesion molecules, Th1 cytokines, and surface receptors regulates T-cell homing and infiltration (101). Tumor cells, in concert with the endothelium, interfere with T-cell infiltration through a variety of molecular mechanisms, including the downregulation of endothelial adhesion molecules and the expression of T-cell inhibitory ligands (102).

Although complement elements can directly act on the endothelium (103–105), little is known about its contribution to the biology of tumor-associated endothelial cells. In the context of adoptive T-cell transfer, the capacity of tumor-reactive CD4^+^ and CD8^+^ T cells to infiltrate tumors requires the local production of C3, complement activation and release of C5a (106). Accordingly, the abrogation of CD55 expression enhances tumor T-cell infiltration (106). Radiotherapy has been found to upregulate the release of C3a and C5a within the irradiated tumors, leading to pronounced infiltration of CD8^+^ effector T cells (107). The presence of C3d on melanoma cells yields greater infiltration by CD4^+^ and CD8^+^ lymphocytes (75). However, in apparent contradiction to these findings, blocking of C3aR or C5aR1 in most cancer models has been associated with an increased infiltration of T cells both in the primary tumor (11, 13, 42, 99, 100, 108, 109) and the metastatic niche (98, 109). The mechanisms underlying this outcome are not fully understood but may be related to the downregulation of immunosuppressive cell populations, such as MDSCs, able to impair T-cell trafficking (11, 12). Complement inhibition also reduces the levels of VEGF (110), which may normalize the tumor vasculature, increasing the infiltration of lymphocytes into tumors (111).

### Modulation of Cytotoxic T-Cell Activity by Complement

An immunosuppressive microenvironment hampers the killing capacity of cytotoxic CD8^+^ T-cells. The pro-tumorigenic effect of complement activity is mediated, in an important way, by promoting immunosuppressing responses within the tumor microenvironment. The critical contribution of complement to regulating immunosuppressive cell populations, such as TAMs, MDSCs, or Tregs, has been recently reviewed (47). C5a acts as a potent chemoattractant for polymorphonuclear MDSCs and stimulates the production of immunosuppressive reactive oxygen and nitrogen species by tumor infiltrating monocytic MDSCs (11). Accordingly, pharmacological blockade of C5aR1 decreases the frequency of MDSCs and impairs tumor growth (11–13, 112). C5aR1 inhibition also downregulates the expression of immunosuppression-related genes within the tumor milieu (12). In addition, C5a contributes to conditioning the premetastatic niche through TGF-β and IL-10-mediated accumulation of Tregs, proliferation of resident alveolar macrophages, and decrease in number and maturation of dendritic cells (98). As a consequence, effector CD4^+^ T-cell responses skew toward a Th2 phenotype, limiting Th1 responses (98, 113).

C5a also affects the biology of macrophages. C5a skews macrophage polarization toward an M2 phenotype via C5aR1 signaling upon *Leishmania* infection (114). After *ex vivo* challenge of human whole blood with heat-killed *Pseudomonas aeruginosa*, C5a induces PD-L1 expression on monocytes, and the production of IL-10 and TGF-β (115). Elevation of PD-L1 expression has also been reported after C1q-mediated polarization of macrophages (89). M2 tumor-associated macrophages (TAMs) are an essential component of the tumor microenvironment that contribute to tumor progression by blocking CD8^+^ T-cell responses (116). Recruitment of tumor-promoting TAMs with a M2-like phenotype is also observed in mouse sarcomas induced in a PTX3-deficient context and characterized by an increase in C5a and CCL2 (44). C5a also promotes hepatic metastases of colon cancer associated with an increase of monocyte chemoattractant protein-1 (MCP1), anti-inflammatory modulators such as arginase-1, IL-10, or TGF-β, and M2-like macrophages (117, 118). Similarly, in a model of squamous carcinogenesis, C5a regulates the protumorogenic properties of C5aR1-expressing mast cells and macrophages, leading to hampered antitumor CD8^+^ T-cell responses (100). A combined treatment with cytotoxic chemotherapy and the blockade of C5aR1 synergistically inhibits the recruitment of effector memory CD8^+^ T cells by both the modification of macrophage- and IFNγ-dependent mechanisms (100). Interestingly, this study suggests that C5a is not generated in the tumors through C3 activation, although further studies are needed to rule out this possibility (119).

Complement C3 activation fragments can also precondition the tumor microenvironment toward immunosuppression. The C3 degradation product iC3b promotes the development of MDSCs *in vitro* (120). Inhibition of complement C3 abrogates the suppressor phenotype of polymorphonuclear MDSCs in the ovarian tumor microenvironment (121). Deletion of C3 in tumor cells also inhibits M2 polarization (122). Signaling mediated by C3a contributes to melanoma tumorigenesis by inhibiting neutrophil and CD4^+^ T-cell responses (108). Interestingly, some studies have suggested a direct effect of complement effectors in the functionality of T cells. C3 inhibits IL-10-mediated cytotoxic properties of tumor-infiltrating CD8^+^ T lymphocytes in an autocrine manner, enhancing melanoma and breast cancer growth (123). Alterations in CD4^+^ T cells by C3/C5-dependent pathways may also have a major role in lung cancer progression (109).

Finally, complement can also slow down the feeding of the cancer-immunity cycle by dying cancer cells. Ribosomal protein S19 (RPS19), upon release from dying tumor cells, interacts with C5aR1 expressed on MDSCs, promoting its recruitment to tumors, the generation of Tregs, the production of immunosuppressive cytokines (including TGF-β), and the reduction of CD8^+^ T-cell tumor infiltration (99).

Overall, tumor-associated complement activation deeply influences the tumor microenvironment, leading to an immunosuppressive state and the attenuation of tumor-specific cytotoxic T-cell responses.

## Complementing the Cancer-Immunity Cycle

**Figure 1:**
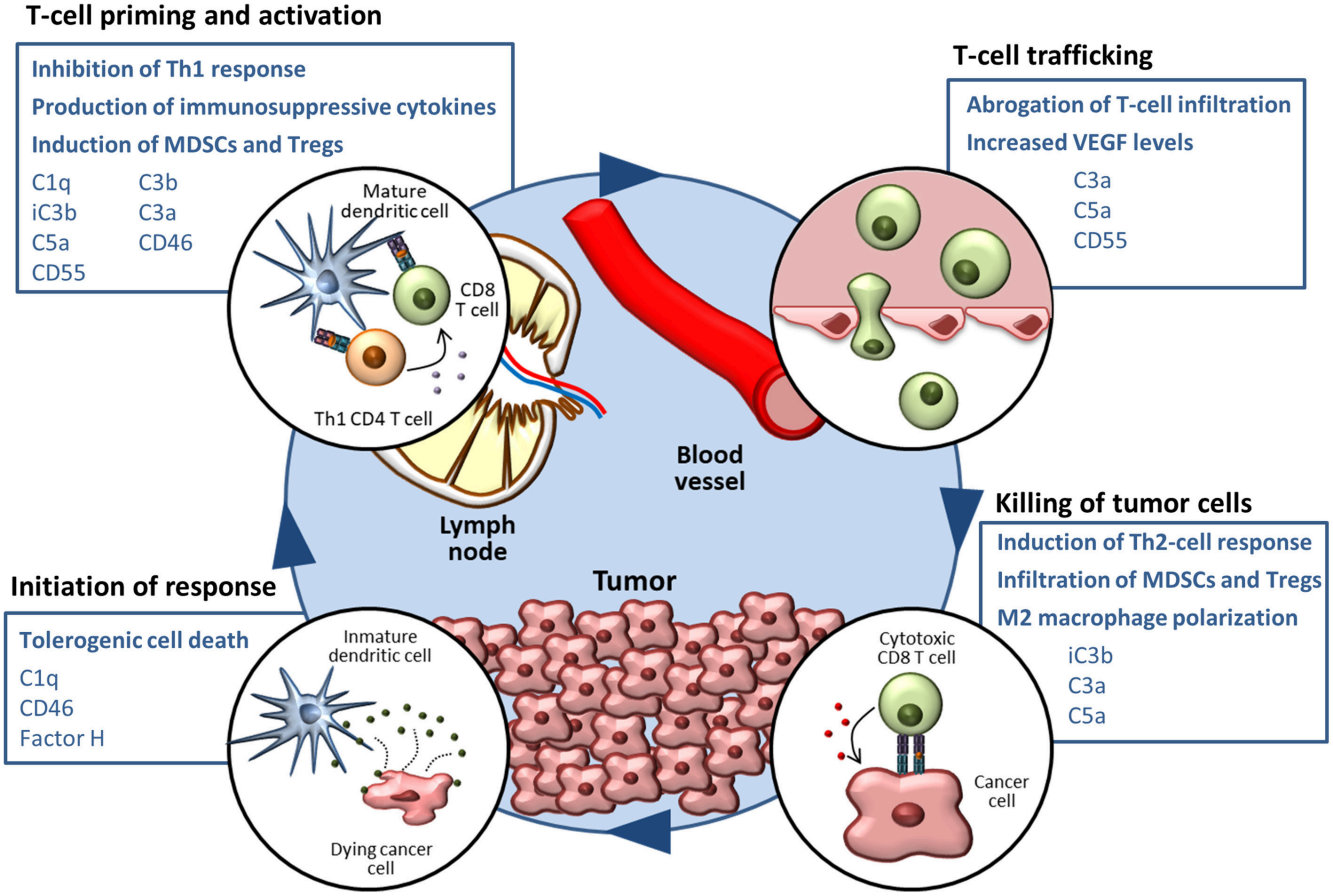
The complement system in the cancer-immunity cycle. The cancer-immunity cycle is summarized in four steps. Complement-mediated mechanisms associated with the inhibition of the cancer-immunity cycle, together with complement components that participate in these processes, are shown in blue boxes.

As reviewed in the previous section, a growing body of evidence supports the notion that complement activities support cancer growth and metastasis in the context of established tumors (124). Many mechanisms related to immune escape and resistance to checkpoint inhibitors can be modulated by elements of the complement system (summarized in Figure 1). The non-immunology-related effects of complement on cancer cell biology, including cancer cell proliferation, survival and invasion capacity (42, 43, 117, 125–137), further reinforces the impact of complement activation in cancer progression.

Based on the regulatory functions of complement in the cancer-immunity cycle, we sought to evaluate whether complement inhibition may represent an effective target for combined immunotherapies in preclinical syngeneic models of cancer. Clinical successes and limitations of anti-PD-1/PD-L1 monotherapy prompted us to use this target as the primary building block for the combination. The C5a/C5aR1 axis was selected as the complement-related target based on the abundant evidence supporting the role of this pathway in the establishment of an immunosuppressive microenvironment (Table 2) (45). Using different lung cancer models, we observed a remarkable synergistic control of lung tumor burden and metastatic progression in animals simultaneously treated with an aptamer against C5a (AON-D21) and an anti-PD-1 monoclonal antibody (13). This effect is accompanied by a negative association between the frequency of CD8^+^ T cells and the presence of MDSCs within tumors, and by a reduction of CD8^+^ T-cell exhaustion markers (13). The synergistic benefit of this combination was later confirmed in models of melanoma and colon cancer (138). Interestingly, PD-1/PD-L1 antibodies induce the production of C5a (138), establishing a regulatory loop between both pathways.

**Table 2:** Contribution of some elements of the complement system to the inhibition of the cancer-immunity cycle.

**Entity**	**Role**	**Affected cancer-immunity step**
C1q	Tolerogenic clearance of dying tumor cells	Initiation of anti-tumor immunity
	Inhibition of antitumor Th1 response	T-cell priming and activation
C3 fragments (C3b, iC3b, C3a)	Tolerogenic clearance of dying tumor cells	Initiation of anti-tumor immunity
	Inhibition of antitumor Th1 response	T-cell priming and activation
	Abrogation of T-cell infiltration	T-cell trafficking
	Differentiation of MDSCs	Killing of cancer cells
	Impaired T-cell cytotoxicity	Killing of cancer cells
C5a	Inhibition of antitumor Th1 response	T-cell priming and activation
	Abrogation of T-cell infiltration	T-cell trafficking
	Angiogenesis	T-cell trafficking
	Tumor infiltration of MDSC and Tregs	Killing of cancer cells
	Polarization toward an M2 phenotype	Killing of cancer cells
	Impaired T-cell cytotoxicity	Killing of cancer cells

**Figure 2:**
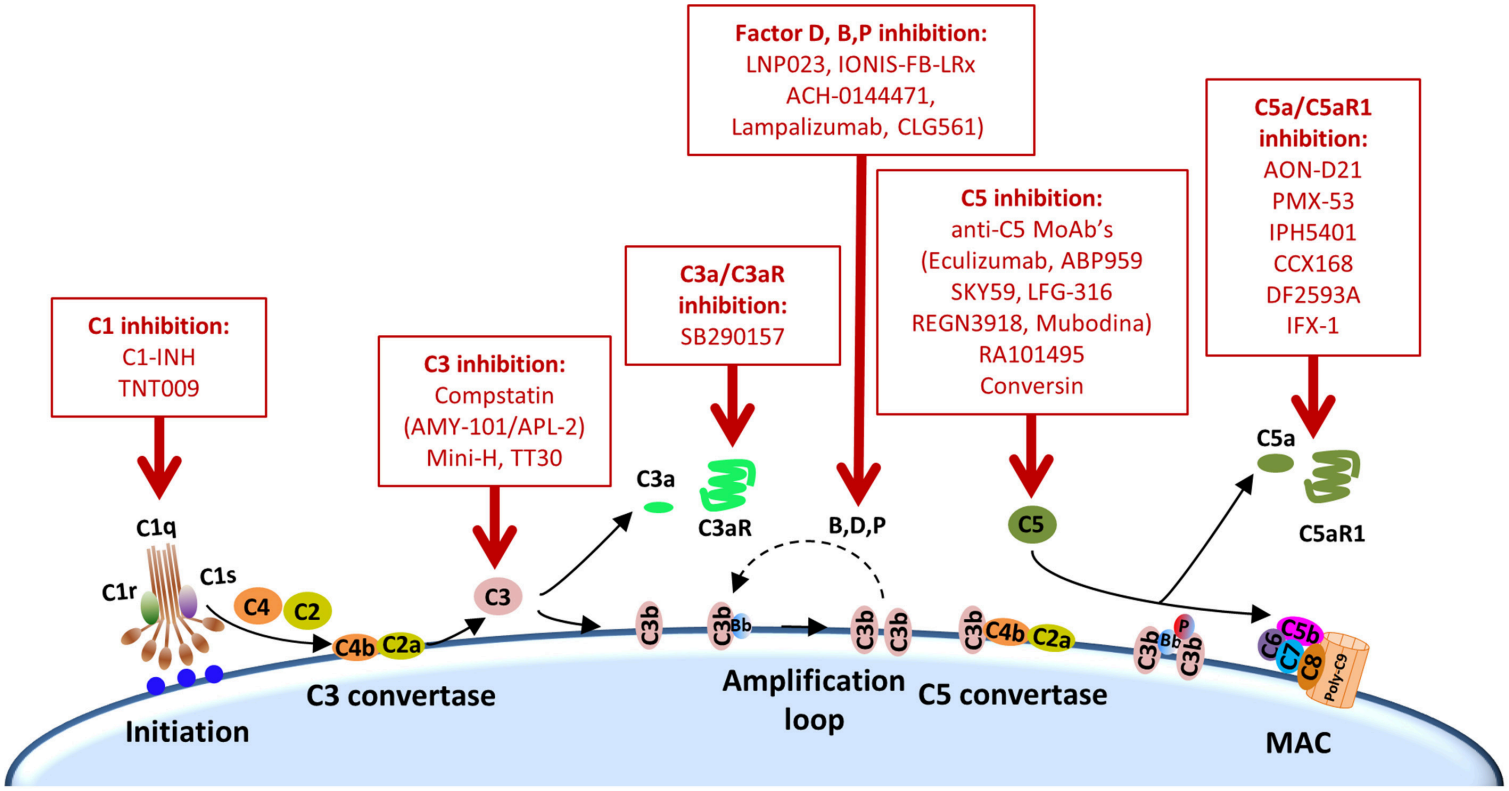
Points of complement inhibition. Steps of the classical complement activation pathway and some inhibitors available for targeting these steps are shown. These points of therapeutic intervention may render synergistic antitumor activities in combination with anti-PD-1/PD-L1 therapies.

Other complement elements, such as C1q, C3, or C3a, may be also targeted to re-educate the tumor microenvironment and sensitize it to the subsequent administration of immune checkpoint blockers (Table 2). A multifaceted repertoire of therapeutic inhibitors targeting these complement elements has been developed, and are currently in preclinical or clinical development (139, 140). Figure 2 shows examples of compounds that may be used to target complement in the context of cancer immunotherapy. Complement C3, the centerpiece of complement activation, represents a particularly attractive target for therapeutic complement inhibition (141). Nonresponsive patients to PD-1/PD-L1 blockade frequently have noninflamed tumors with a defect in the early stages of the cancer-immunity cycle (15). Opsonization of dying cells with C3 fragments induces the production of anti-inflammatory cytokines and reduces the costimulatory molecules needed for the maturation of dendritic cells, resulting in T-cell tolerance (90). Therefore, C3 blockade may have a beneficial impact in the early stages of the cancer-immunity cycle, converting a noninflamed tumor into an inflamed tumor susceptible to PD-1/PD-L1 blockade. Moreover, both C3a and C5a production would be impaired (at least in the case of inhibitors, such as compstatin, that blocks C3 activation by all pathways). Interestingly, a simultaneous blockade of C3aR and C5aR1 has been reported to enhance the efficacy of anti-PD-1 therapy against melanoma cells (123). Deletion of C3 in tumor cells that had high C3 expression enhanced efficacy of anti–PD-L1 treatment (122). Additionally, complement C3 inhibition may have antitumor potential in the context of other immune combinations. For example, C3 inhibition by complement depletion or the use of the inhibitor compstatin enhances the antitumor efficacy of oncolytic virus (142, 143) and induces natural killer (NK)-mediated antitumoral responses (144). Inhibition of complement activation upstream of all complement effectors also appears to be a rational approach. In this sense, combinatorial therapies involving inhibitors of C1q (e.g., C1-INH), which presents both complement-dependent and -independent tumor promoting activities, merit further investigation. Finally, it has to be noted that most complement inhibitors target the extracellular complement system, preserving its intracellular activity. This may be of upmost importance, since intracellular C3aR and C5aR1 signaling pathways seem to be required for T-cell survival (28).

Finally, it is interesting to point that the preclinical findings showing the feasibility and value of blocking C5a/C5aR1 to increase tumor-killing efficacy of checkpoint inhibitors have been the basis for the design of a phase I/II study (STELLAR-001). In this trial, the safety and efficacy of durvalumab (an anti-PD-L1 monoclonal antibody) is being tested in combination with IPH5401 (an anti-C5aR1 monoclonal antibody) in patients with selected solid tumors, including non-small cell lung cancer and hepatocellular carcinoma (NCT03665129). We are looking forward to the outcome of this trial, as well as to the clinical evaluation of novel combinations involving complement inhibitors.

## Concluding Remarks

This is an exciting time for the complement field, in which new biological concepts have brought new therapeutic opportunities. Based on the extensive literature associating complement activation and cancer progression, we propose here that substantial clinical benefits can be achieved by multi-modal anticancer immunotherapies targeting both complement-mediated mechanisms (to reverse immunosuppression), and PD-1/PD-L1 immune checkpoints (to re-activate T-cell functionality). Our preclinical studies supporting the idea that C5a/C5aR1 inhibition creates a “window of opportunity” for the administration of anti-PD-1/PD-L1 checkpoint inhibitors pave the way for the evaluation of other complement-based combinations. The challenge being that many potential combinations can be evaluated. Insights into how complement switches from tumor suppressing to tumor promoting activities at the onset of disease, as well as how to manage this dichotomy should be important research areas in order to establish the best therapeutic strategies. The differences between mice and humans in complement-mediated T-cell responses should also be considered (28, 87). To overcome this limitation, faithful mouse models that recapitulate the complexity of the human immune context in the tumor microenvironment are urgently needed (145).

## Author Contributions

RP and JL designed the concept. All authors wrote the manuscript. SO-E prepared the figures. All authors read and approved the final version of the manuscript.

### Conflict of Interest Statement

JL is the founder of Amyndas Pharmaceuticals, which is developing complement inhibitors (including third-generation compstatin analogs such as AMY-101) and inventor of patents or patent applications that describe the use of complement inhibitors for therapeutic purposes, some of which are developed by Amyndas Pharmaceuticals. JL is also the inventor of the compstatin technology licensed to Apellis Pharmaceuticals (i.e., 4(1MeW)7W/POT-4/APL-1 and PEGylated derivatives). AM is inventor of patents related to PTX3 and gets royalties from related immunoassays. RP and DA are inventors of patents related to the use of complement C4 fragments as diagnostic and prognostic cancer biomarkers. The remaining author declares that the research was conducted in the absence of any commercial or financial relationships that could be construed as a potential conflict of interest.
